# Working memory of school-aged children on the autism spectrum: Predictors for longitudinal growth

**DOI:** 10.1177/13623613231165599

**Published:** 2023-04-21

**Authors:** Sohyun An Kim, Connie Kasari

**Affiliations:** University of California, Los Angeles, USA

**Keywords:** approaches to learning, autism, longitudinal development, socioeconomic status, student–teacher relationship, working memory

## Abstract

Working memory is an important component of executive functioning, an area of difficulty for many autistic children. However, executive functioning and working memory are highly malleable throughout childhood, and various student-level and environmental factors play important roles in their development. This study used the Early Childhood Longitudinal Studies–Kindergarten Class of 2011. Conditional latent growth models were constructed to identify possible predictors for autistic children’s working memory performance upon entering kindergarten and their relative growth throughout their elementary school years. Study results indicate that socioeconomic status and students’ approaches to learning were positively associated with autistic children’s working memory performance upon school entry. Students’ approaches to learning positively predicted their rate of growth during the first 3 years and negatively predicted their rate of growth during the last 3 years of elementary school. Student–teacher relationship was positively associated with the rate of growth during the last 3 years of their elementary school years. Furthermore, autistic students who started at a lower standing in working memory upon school entry were more likely to receive special education services during their elementary school years. Practical and policy implications as well as future directions are discussed.

**Lay abstract:**

Working memory is an important skill for school success, and it involves holding information in our memory while using it to solve complex problems at the same time. Autistic children often have difficulty with working memory. Because working memory development can be easily influenced by many factors from a young age, it is important to find factors that help with autistic children’s development. This study tested the factors that are related to autistic children’s working memory when they start kindergarten and the factors that can help with rapid improvement throughout their elementary school. We used a nationally representative data set that followed the same group of children from kindergarten to fifth grade. We found that autistic students from backgrounds with more resources and students with advanced learning approaches such as being organized, being excited to learn, and paying careful attention to their work, started school with strong working memory. Autistic students with advanced learning approaches continued to make rapid improvements during the first 3 years, and then their growth slowed down during the last 3 years. Autistic students who had a good relationship with their teachers made rapid improvements during the last 3 years of their elementary school. In addition, autistic children who struggled with working memory upon school entry were more likely to receive special education services at school. These findings suggest that we need effective ways to teach young autistic children important learning-related behaviors from a very young age through the school system, and teachers must prioritize building positive relationships with their students.

Working memory, an important component of executive functioning (EF), involves maintaining and updating incoming information for relevance to the current task, and then revising and replacing old information with newer, more relevant information (Garon et al., 2008; Miyake et al., 2000; Morris & Jones, 1990). Working memory begins to develop early in life and shows consistent growth through the adolescent years (Conklin et al., 2007; Gathercole et al., 2004; Luciana et al., 2005). Working memory is found to be associated with various school outcomes such as early literacy skills (Rojas-Barahona et al., 2015), mathematical skills (Bull et al., 2008; Harvey & Miller, 2017), social and emotional (Roughan & Hadwin, 2011; Schmeichel et al., 2008), and behavioral readiness for school (Schoemaker et al., 2013). As such, working memory has been known to play a critical role in cognitive development and school success.

## Predictors for growth in working memory for children on the autism spectrum

It is well-established in the literature base that children on the autism spectrum are more likely to experience greater challenges with their working memory development (Andersen et al., 2015; Chen et al., 2016; Happé et al., 2006). EF development is extremely sensitive to environmental factors, and EF can be improved from a very young age into adulthood (e.g. Cannon et al., 2011; Diamond & Lee, 2011; Hughes, 2011). Therefore, it is critical that researchers examine possible environmental-level factors such as family or school environment and student-level factors such as learning-related behaviors that can contribute to autistic children’s growth in working memory during their elementary school years.

### Socioeconomic status

A family’s high socioeconomic status (SES) is often linked to higher working memory in children (Hackman et al., 2015; John et al., 2019). More specifically, SES disparity in working memory is persistent from a very young age into middle childhood (Hackman et al., 2015), and even into early adulthood (Last et al., 2018). Moreover, EF skills including working memory-related tasks in young children mediate the longitudinal relationship between their socioeconomic risk status and school readiness (Perry et al., 2018).

While it is well-established that the SES is a critical factor that can influence children’s working memory concurrently, less is known about the longitudinal relationship between SES and autistic children’s rate of growth in working memory. Therefore, a closer examination of the relationship between SES and the developmental trajectory of working memory in children with autism is indicated.

### Special education services and interventions for autism

While there are documented delays in working memory in autistic children, what remains unclear is the effectiveness of school-based intervention services in remediating autistic children’s working memory. There is emerging evidence on the effectiveness of school-based EF interventions that directly target EF skills for autistic as well as for neurotypical children (Cavalli et al., 2022; Otero et al., 2014), but generalizability, availability, and feasibility of these interventions remain as barriers for a wider population.

School-aged children with autism in the United States are entitled to various special education services as per the federal Individuals with Disabilities Education Act (IDEA). Fourteen percent of all public school students receive special education services under the IDEA, and 11% of these students qualify for the special education services for their autism diagnosis (Individuals with Disabilities Education Act [IDEA] database, 2020). These services are intended to assist children with disabilities so that they may benefit from their public education experience and to improve educational results by meeting their unique needs and providing necessary tools (IDEA, 2004). These special education services may directly or indirectly target working memory skills as part of their educational interventions.

While little is known about the relationship between special education services that autistic children are eligible for and their working memory development, a school-based intervention that directly targets EF skills (Unstuck and On Target) was found to improve autistic children’s EF skills (Cannon et al., 2011). Therefore, it will be worthwhile to investigate if special education services have any effect on their working memory development as it is an important predictor for overall school success for school-aged children.

### Student–teacher relationship

One of the factors that appears critical to children’s positive school outcomes is the student–teacher relationship (STR). High-quality STR involves creating close and supportive interactions within the classroom, while teachers are responsive to students’ individual needs and students’ autonomy and perspectives are valued (Pianta et al., 2012). Quality of STR plays an important role in children’s academic engagement, especially for those children with learning difficulties (Roorda et al., 2011). Not surprisingly, working memory is found to be positively related to STR in young neurotypical children (Vandenbroucke et al., 2018). It was also found that children’s EF skills including working memory prior to school entry positively predicted STR in kindergarten (McKinnon & Blair, 2018), and positive STR was found to be a consistent predictor for EF development in children (Cumming et al., 2020). Moreover, poorer EF was associated with lower teacher-rated kindergarten readiness for children with poor STR quality (Graziano et al., 2016).

Importantly, children with autism may be at greater risk for developing poor STR. Teachers report having a more difficult time building positive relationships with children with autism when compared to students with intellectual disability or typical development (Blacher et al., 2014). Based on the evidence that links children’s working memory and STR, positive STR may play a critical role in autistic children’s working memory development, and close examination of such relationships is essential in fostering school success in autistic children.

### Approaches to learning

Approaches to learning (ATL) refers to a select set of positive learning behaviors such as keeping belongings organized, showing eagerness to learn new things, working independently, adapting to changes in routine easily, persisting in completing tasks, paying attention well, and following classroom rules (Tourangeau et al., 2019). Evidence suggests that ATL is closely linked to children’s school readiness (Lee, 2012; Vitiello & Greenfield, 2017). Furthermore, children’s early ATL is found to be associated with their long-term school outcomes, especially in reading and math regardless of particular demographic characteristics (Li-Grining et al., 2010).

Skills related to ATL appear to be closely associated with EF skills (Vitiello & Greenfield, 2017). More specifically, poor working memory is found to contribute to poor organizational and planning skills (Kofler et al., 2018), and children’s overall EF skills are often linked to their attentiveness, following rules in group settings and other behavioral and socioemotional skills that are necessary for their school readiness (Mann et al., 2017; Pellicano et al., 2017). Such a close relationship between ATL and working memory (WM) implies that these two skills may exert bidirectional influences. In other words, while research shows that WM plays an important role in the development of skills related to ATL, it would be also worthwhile to investigate whether children who are equipped with advanced ATL upon school entry can consequently make greater gains in WM throughout their school years. Taken together, ATL is an important factor to be investigated when examining autistic children’s developmental trajectories of working memory.

## Gap in literature

While many studies have examined predictors for children’s working memory at one point in time, far less is known about contributing factors for the rate of growth in working memory during children’s elementary school years. Predictors for working memory development for neurotypical children have been well-explored (e.g. Hackman et al., 2015; Vandenbroucke et al., 2018), but we know little about growth in working memory development over the entirety of the elementary school years for the autistic population specifically despite well-documented delays in their WM. Therefore, this study aims to take a close examination of autistic children’s student- and environmental-level factors that are associated with their working memory performance upon school entry and the factors that contribute to their rate of growth throughout their elementary school years. We hypothesized that higher family SES and students’ advanced ATL would positively predict autistic children’s working memory upon school entry, and STR, ATL, and special education services at school would foster greater longitudinal growth in working memory during their elementary school years.

## Method

### Data set

This study used the restricted version of the Early Childhood Longitudinal Study–Kindergarten Class of 2010–2011 (ECLS-K:2011) data set. ECLS-K:2011 data set is a nationally representative data set, which follows the same cohort of children from kindergarten through fifth grade (Tourangeau et al., 2019). Data were collected from Fall of kindergarten in year 2010 (T1) through Spring of fifth grade in year 2016 (T9), across nine time points (Table 1).

**Table 1.** Data collection schedule from T1 to T9.

	Semester and grade	School year
T1	Fall of kindergarten	2010–2011
T2	Spring of kindergarten	
T3	Fall of first grade	2011–2012
T4	Spring of first grade	
T5	Fall of second grade	2012–2013
T6	Spring of second grade	
T7	Spring of third grade	2014
T8	Spring of fourth grade	2015
T9	Spring of fifth grade	2016

Source: US Department of Education, National Center for Education Statistics (NCES), The Early Childhood Longitudinal Study–Kindergarten Class of 2010–2011 (ECLS-K:2011). Restricted-use data files.

### Participants

In total, approximately 18,170 children participated in the data collection. Students were included in the current sample in this study if: (1) parents responded at least once during the six rounds of interview that their child had a diagnosis of autism or (2) the special education teacher responded at least once that the child was receiving special education services for a diagnosis of autism. Approximately (*N* ≈ 310) students were identified as having autism and were included in the analysis. Sample sizes reported in this study are rounded to the nearest 10 per confidentiality agreement.

### Measures

#### Demographic characteristics

Demographic characteristics of the sample included (1) race/ethnicity (White, Black, Hispanic, Asian-American/Pacific Islanders/Native Americans (AAPINA), other) and (2) sex assigned at birth (male, female), (3) income range, and (4) parent’s educational level.

#### Variables

##### Working memory

Number Reversed subset of the Woodcock-Johnson III (WJ III: Woodcock et al., 2001) was administered to measure auditory working memory from kindergarten through fifth grade, across nine time points. Students were asked to repeat verbally presented strings of numbers backwards, starting with two-number sequences. The length of sequence increased after five trials, up to a maximum of eight numbers. If the child responded incorrectly for three consecutive trials, the task ended instead of progressing to a longer number sequence. Each item was scored as “correct,” “incorrect,” or “not administered” (Tourangeau et al., 2019).

For the purposes of this study, the *W* score for the Number Reversed subtask was used. The *W* score is a standardized equal-interval score that represents both a child’s ability and the item difficulty. It is particularly suited for longitudinal analyses, regression, and correlation (Tourangeau et al., 2019).

##### Sex assigned at birth

A variable for students’ sex was drawn from parent-reported information about the child’s sex or the school’s administrative records.

##### Race

A variable for students’ race was drawn from either the parent-reported information about the child’s race or the school’s administrative records. The administrative records were used only if parent responses about the child’s race were missing.

##### SES

Socioeconomic status was computed three times during the data collection period, at T1, T4, and T9. It was computed using responses from the parent interview. The five components used to create the SES variable were (1) parent one’s education, (2) parent two’s education, (3) parent one’s occupation, (4) parent two’s occupation, and (5) household income. A single mean score was then calculated from these three time points for each child.

##### Attention deficit hyperactive disorder

In T2, T4, T6, T7, T8, and T9, parents were asked during the parent interview, “Did you obtain a diagnosis of a problem from a professional?” If the response was yes, they were asked a follow-up question to specify what the diagnosis was. One of the options was *Attention Deficit Hyperactive Disorder* (*ADHD*). The ADHD variable was assigned a value of 1 if parents responded at least once during the six rounds of interview that the child had a diagnosis of ADHD. If not, 0 was assigned.

##### Learning disability

In T2, T4, T6, T7, T8, and T9, parents were asked during the parent interview, “Did you obtain a diagnosis of a problem from a professional?” If the response was yes, they were asked a follow-up question to specify what the diagnosis was. One of the options was *specific learning disabilities.* The Learning Disability (LD) variable was assigned a value of 1 if parents responded at least once during the six rounds of interview that the child had a diagnosis of specific LD. If not, 0 was assigned.

##### Special education services for autism

In T2, T4, T6, T7, T8, and T9, special education teachers were asked, “For which of the following disabilities has this child received special education or related services this school year, whether for the child’s primary disability or another of his or her disabilities?” Special Education Services for Autism variable was assigned a value of 1 if teachers chose “Autism” to this question at least once during the six rounds of interview. If not, 0 was assigned.

##### STR

The *Student–Teacher Relationship Scale* (*STRS*) (Pianta & Stuhlman, 2004) was used in T2, T4, T6, and T7 to assess teacher-reported measure of closeness and conflict between teacher and child. The STRS contains two scales: Closeness and Conflict. The Closeness and Conflict scales are not only theoretically closely related but they are also negatively correlated each round, with correlations ranging from −0.34 to −0.51 (*p* < 0.05). Considering the fact that many evidence-based programs that are designed to foster positive STR (e.g. Establish-Maintain, Restore, the BRIDGE program) focus on strategies that establish positive and trusting relationships such as praise, 1:1 time, expressing care, and respect (Kincade et al., 2020) as opposed to resolving conflicts, it was decided that the Closeness scale was more in line with the aforementioned STR-building programs theoretically. Therefore, the Closeness scale was used in the current analysis. The Closeness scale contained seven items and measured the affection, warmth, and open communication that the teacher experiences with the child. Each item was scored on a 5-point scale ranging from “definitely does not apply” (1) to “definitely applies” (5). The average score for each time point was computed when the teacher provided a rating on at least five of the seven items included in the scale (Tourangeau et al., 2019).

One composite score for the Closeness scale score was computed for each child by averaging the scale scores from the four time points (T2, T4, T6, T7). The average score was computed when the child had at least two or more scale scores from the four time points.

##### ATL

The child-level questionnaire was administered at T1, T2, T4, T5, T7, T8, and T9, and was completed by the child’s teacher. Teachers were asked to rate the frequency of the following behaviors of the child: (1) keeps belongings organized, (2) shows eagerness to learn new things, (3) works independently, (4) easily adapts to changes in routine, (5) persists in completing tasks, (6) pays attention well, and (7) follows classroom rules. Response options included 1—“Never,” 2—“Sometimes,” 3—“Often,” and 4—“Very Often.” The ATL scale score was then created for each time point by computing the mean of the seven items for each time point. A score was computed when the teachers provided a rating on at least four of the seven questions above. A single mean composite score was then created for each child by averaging the scores from those seven time points if the child had scores from at least four of the seven time points.

### Analyses

#### Missing data

Missingness in the working memory scores at each time point is summarized in Table 2. T3 and T5 were excluded from current analyses due to high level of missingness (>70%).

**Table 2.** Percentage of missingness in the working memory variable at each time point.

Time point	% of missingness
	Autism sample (*N* = 310)
T1	30.19%
T2	14.61%
T3^a^	73.38%
T4	21.43%
T5^a^	73.70%
T6	24.68%
T7	29.22%
T8	33.77%
T9	38.64%

Source: US Department of Education, National Center for Education Statistics (NCES), The Early Childhood Longitudinal Study–Kindergarten Class of 2010–2011 (ECLS-K:2011). Restricted-use data files.

a Excluded from the analysis due to high percentage of missingness.

Little’s test of missing completely at random (MCAR) (Little, 1988) was conducted after excluding T3 and T5 using SPSS version 28. The current sample passed Little’s MCAR test with *χ*^2^ = 133.860 (*p* = 0.584). Therefore, missing data were handled by Full Information Maximum Likelihood (FIML) as this method is shown to be robust with structural equation models (SEMs) under the assumption of MAR (missing-at-random) (Allison, 2003).

#### Model evaluation criteria for latent growth model

This study adopted the following evaluation criteria for the maximum likelihood (ML) method in structural equation modeling: comparative fit index (CFI) ⩾ 0.95; standardized root mean square residual (SRMR) ⩽ 0.08; root mean square error of approximation (RMSEA) ⩽ 0.06 (Hu & Bentler, 1999).

#### Conditional latent growth model

In order to determine the best fitting latent growth model (LGM) for the working memory development, a linear model was built first using Lavaan package (Rosseel, 2012) in R. The starting point (i.e. intercept) and rates of growth (i.e. slopes) were modeled as latent variables. Loadings for the latent intercept were fixed to one, and loadings for the latent slope started at zero for the first time point, and then increase by one for subsequent loadings. Working memory was assessed throughout nine time points in the data set, and each time point except for Fall of first grade (T3) and Fall of second grade (T5) served as a manifest variable in the model. Each whole number increment reflected one semester. If the assessments were done 1 year apart (e.g. Spring fourth grade and Spring fifth grade), the loading increased by two, which reflects twice as much time passing between data collection periods. This model (Model 1) demonstrated a poor fit, with the fit indices far below the aforementioned criteria (*χ*^2^ = 178.576, degree of freedom (df) = 63, CFI = 0.888, RMSEA = 0.093, SRMR = 0.069).

**Figure 1.**
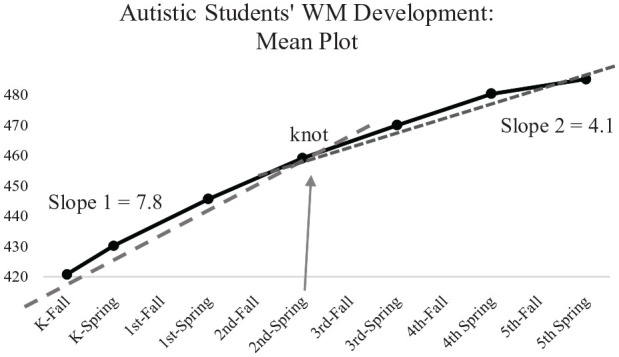
Mean plot of the sample’s WM development, slopes, and the knot. Source: US Department of Education, National Center for Education Statistics (NCES), The Early Childhood Longitudinal Study–Kindergarten Class of 2010–2011 (ECLS-K:2011). Restricted-use data files.

In order to improve the model fit, piecewise LGM was built. Piecewise linear models are a flexible and parsimonious approach to estimate nonlinear longitudinal trajectories with two linear slope factors (Flora, 2008). With piecewise models, a decision needs to be made for the transition point or a “knot” representing a time point when the two linear slopes meet. To estimate the optimal location of the “knot,” sample means for each time point were plotted. Upon visual examination of the WM trajectory of the autism sample, either T6 or T7 appeared to be a good location for the knot. Therefore, two piecewise models were developed with a knot at T6 and with a knot at T7 (Model 2 and Model 3, respectively). Both models improved the fit indices significantly as compared to the linear model. The fit indices were *χ*^2^ = 81.846, df = 51, CFI = 0.970, RMSEA = 0.053, SRMR = 0.033 for Model 2 and *χ*^2^ = 96.466, df = 51, CFI = 0.956, RMSEA = 0.065, SRMR = 0.045 for Model 3. Because Model 2 met the model evaluation criteria more closely, this model was chosen as the final model. Therefore, the “knot” for the piecewise model was identified at Spring of second grade (T6). These fit indices are summarized in Table 3. Figure 1 illustrates the mean plot of the sample’s WM development, the two slopes, and the location of the knot.

**Table 3.** Model selection process.

Working memory	Model type	*χ* ^2^	df	*p*	CFI	RMSEA	SRMR
Model 1	Linear	178.576	63	0.000	0.888	0.093	0.069
Model 2^a^	Piecewise; knot at T6	81.846	51	0.004	0.970	0.053	0.033
Model 3	Piecewise; knot at T7	96.466	51	0.000	0.956	0.065	0.045

Source: US Department of Education, National Center for Education Statistics (NCES), The Early Childhood Longitudinal Study–Kindergarten Class of 2010–2011 (ECLS-K:2011). Restricted-use data files.

df: degree of freedom; CFI: comparative fit index; RMSEA: root mean square error of approximation; SRMR: standardized root mean square residual.

a Final model.

##### Multivariate normality

Mardia’s skewness and kurtosis test was conducted to test the assumption of multivariate normality (Kres, 1983; Mardia, 1980). The set of variables included in the final model met the assumption of multivariate normality (*p* > 0.05).

##### Covariates

The following covariates entered the model for controlling purposes: *Sex Assigned at Birth, Race, ADHD status, and LD status.* The following covariates entered the model as time-invariant covariates to test the predicting power on the parameter estimates: *SES, Special Education Services for Autism, STR*, and *ATL*. These four variables were selected to enter the LGM model based on their hypothesized relationship with children’s working memory development. Autistic community members were not involved in the study.

## Results

### Demographic characteristics

Demographic characteristics of the sample are illustrated in Table 4. Majority of the children in the sample were male (82%). More than half of the children’s parents (62%) attended 2- to 4-year colleges, and 54% of the children were White.

**Table 4.** Demographic characteristics of the sample.

Demographic characteristics	(*N* = 310)
Race	
White	170 (54%)
Black	20 (8%)
Hispanic	70 (21%)
Asian-American/Pacific Islanders/Native American	40 (11%)
Other	20 (7%)
Sex assigned at birth	
Female	60 (18%)
Male	250 (82%)
Income	
US$20,000 or less	50 (19%)
US $20,000–US$30,000	30 (13%)
US$30,000–US$50,000	50 (20%)
US$50,000–US$75,000	40 (14%)
US$75,000–US$100,000	30 (13%)
US$100,000–US$200,000	40 (16%)
US$200,000 or more	20 (6%)
Parents’ educational level	
High school	50 (24%)
2- to 4-year college	140 (62%)
Postgraduate degree	30 (15%)

Source: US Department of Education, National Center for Education Statistics (NCES), The Early Childhood Longitudinal Study–Kindergarten Class of 2010–2011 (ECLS-K:2011). Restricted-use data files.

*Note*: N rounded to the nearest 10 per confidentiality agreement.

### Predictors

#### SES

Family’s SES was found to predict autistic children’s initial status of working memory performances such that the higher the family’s SES, the higher the children’s initial status at Fall of kindergarten. The relationship was statistically significant (*β* = 0.227, *p* = 0.002). However, the relationship between family’s SES and either Slope 1 or Slope 2 was not statistically significant (Slope 1: *β* = −0.113, *p* = 0.246; Slope 2: *β* = −0.011, *p* = 0.915).

#### Special education services for autism

Having received special education services for autism at school at least at one time point was negatively related to autistic children’s initial status with working memory performances at Fall of kindergarten (*β* = −0.306, *p* < 0.001). Having received special education services for autism at least at one time point did not have a statistically significant relationship with either Slope 1 or Slope 2 (Slope 1: *β* = 0.183, *p* = 0.070; Slope 2: *β* = 0.065, *p* = 0.529).

#### STR

Teacher-reported rating of autistic children’s STR did not have a statistically significant relationship with their initial status at Fall of kindergarten or rate of growth from Fall of kindergarten to Spring of second grade (Slope 1) (*β* = 0.068, *p* = 0.527). However, autistic children’s higher rating on STR was associated with a higher rate of growth from Spring of second grade to Spring of fifth grade, and this relationship was statistically significant (*β* = 0.266, *p* = 0.012).

#### ATL

Teacher-reported rating of autistic children’s ATL was found to predict a higher initial status at Fall of kindergarten (*β* = 0.341, *p* < 0.001) as well as Slope 1 (*β* = 0.242, *p* = 0.021). However, autistic children’s rating on ATL predicted a slower rate of growth beyond Spring of second grade (Slope 2) (*β* = −0.222, *p* = 0.040).

**Figure 2.**
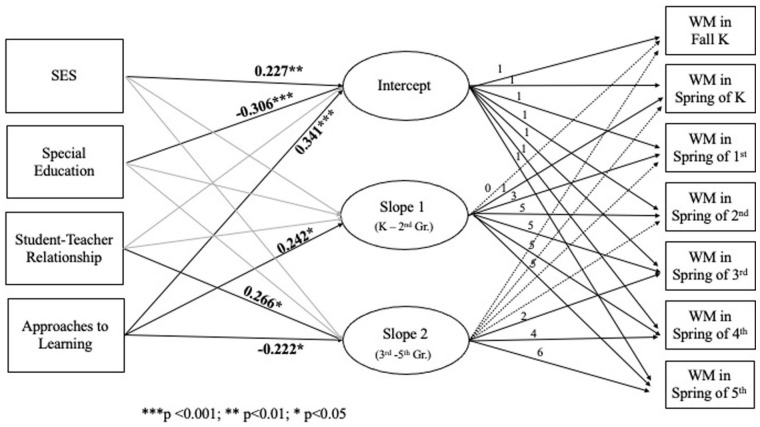
Estimated latent growth model with five covariates predicting autistic children’s initial status on working memory upon school entry, rate of growth from kindergarten to second grade (Slope 1), and rate of growth from third grade to fifth grade (Slope 2). Nonsignificant pathways are gray lines. Whole number increment in factor loadings for the slopes reflects 1 semester. Source: US Department of Education, National Center for Education Statistics (NCES), The Early Childhood Longitudinal Study–Kindergarten Class of 2010–2011 (ECLS-K:2011). Restricted-use data files.

Relationships between the aforementioned predictors and autistic children’s initial status, Slope 1, and Slope 2 of the working memory trajectory are summarized in Table 5. Figure 2 illustrates the LGM of autistic children’s working memory development and covariates predicting the intercept and the slopes.

**Table 5.** Relationships between covariates and autistic children’s developmental trajectory of working memory.

Initial status (Fall K)			Slope 1 (Fall K to Spring 2nd Gr.)		Slope 2 (Fall K to Spring 5th Gr.)	
Covariates	Standardized *β*	*p*	Standardized *β*	*p*	Standardized *β*	*p*
SES	**0.227**	0.002*	−0.113	0.246	−0.011	0.915
Special Education services	**−0.306**	0.000*	0.183	0.070	0.065	0.529
Student–teacher relationship	−0.015	0.857	0.068	0.527	**0.266**	0.012*
Approaches to learning	**0.341**	0.000*	**0.242**	0.021*	**−0.222**	0.040*
Sex (female = 1)	**−0.172**	0.019*	0.035	0.715	0.006	0.950
Race (White = 1)	0.019	0.791	−0.002	0.982	−0.001	0.993
ADHD (ADHD = 1)	0.085	0.266	**0.258**	0.011*	**−0.250**	0.015 *
LD (LD = 1)	−0.127	0.072	−0.029	0.756	0.146	0.129

Source: US Department of Education, National Center for Education Statistics (NCES), The Early Childhood Longitudinal Study–Kindergarten Class of 2010–2011 (ECLS-K:2011). Restricted-use data files.

SES: socioeconomic status; ADHD: attention deficit hyperactive disorder; LD: learning disability.

* *p* < 0.05.

## Discussion

This study explored possible student- and environmental-level factors that may contribute to autistic children’s growth in working memory over time, as well as the associations between their working memory performance upon school entry and these factors.

Broadly, higher family SES was positively related to autistic students’ working memory performances upon school entry. Furthermore, those who started at a lower standing in working memory upon school entry were more likely to receive special education services, and also were more likely to have a lower rating in ATL throughout their elementary school years. Furthermore, students’ ATL was positively related to autistic children’s rate of growth in working memory during the first 3 years and negatively related to their rate of growth during the last 3 years of their elementary school. Better STR was positively associated with their rate of growth during the last 3 years of their elementary school years.

Positive relationship between family’s SES and autistic students’ working memory upon school entry is in line with the existing literature base that supports SES disparities on children’s EF performances from young ages (Bernier et al., 2010; Hackman et al., 2015; John et al., 2019; Lengua et al., 2014). However, SES did not influence the rates of growth throughout their elementary school years. This implies that SES disparity exists upon school entry among autistic children, and the gap in working memory persists without narrowing over time. The persistent SES disparity throughout their childhood indicates that school or other external factors does not compensate for the working memory gap that existed upon entering school. Thus, there may be some external factors prior to entering kindergarten that are attributable to the SES disparity upon entering school. Importantly, Hackman et al. (2015) found that early childhood home environment (i.e. degree of enrichment in the home, including toys and books) partially mediated the SES disparity in working memory. As it is well-established that working memory plays an important role in children’s school success and that autistic children demonstrate challenges in working memory skills, this finding has an important policy implication for autistic children from resource-strapped contexts. One important target for early intervention programs such as the Program for Infants and Toddlers with Disabilities (i.e. Part C of IDEA) for autistic children and their families in the United States should be enriching home environments in order to address such persistent SES disparities in autistic children’s working memory. However, many children with autism do not receive Part C services as they are diagnosed often past age 3 years (Maenner et al., 2020), and such delay is more pronounced among children from low SES backgrounds (Fountain et al., 2011; Mazurek et al., 2014; Parikh et al., 2018). The current finding underscores the importance of all eligible children, especially those from a low SES background, being screened in a timely manner so that they can access early intervention services (i.e. Part C of IDEA) that could potentially be beneficial for their working memory development. In addition, there is an urgent need for future research to further identify environmental factors that can remediate the SES disparity in autistic children’s working memory development.

On a related note, this study found that autistic children with more impaired working memory upon school entry were more likely to receive special education services at school (i.e. Part B of IDEA) during their elementary school years. However, contrary to our hypothesis, special education services did not predict greater longitudinal gains in working memory. There is emerging evidence that school-based interventions that directly target EF domains can have positive effects (Cannon et al., 2011; Cavalli et al., 2022; Otero et al., 2014). Taken together, it is critical that researchers and educators examine ways that these evidence-based interventions can be broadly utilized through the Services for School-Aged Children (i.e. Part B of IDEA), which can potentially facilitate working memory development for autistic children.

Moreover, research shows that ATL is closely related to children’s school readiness (Lee, 2012; Vitiello & Greenfield, 2017) and long-term school success (Li-Grining et al., 2010). The current finding indicates that autistic students with high ATL appear to outperform on working memory tasks than those with lower ATL, continue to make rapid progress during the first 3 years of schooling, and their rate of growth slows down as they reach the maturity point (i.e. a point in the development when such susceptibility decreases and the growth stabilizes). It may be that autistic children who start formal schooling with advanced ATL are at a greater advantage for rapid working memory development in early years as ATL serves as a strong foundation or prerequisite skills. Slower rate of growth during the latter half of their elementary school years may be explained by the stabilization of their growth during that time, attributable to their rapid growth during the early years and therefore reaching their maturity point sooner than those with weaker ATL. On a related note, the literature base indicates that EF is positively associated with skills related to ATL (Vitiello & Greenfield, 2017), and working memory’s role in regulating attention is often linked to organizational and goal-directed behaviors in children with ADHD (Kofler et al., 2018). The current finding corroborates such evidence by indicating a similar association between working memory and skills related to ATL among children on the autism spectrum.

This finding has an important practical implication for parents, early childhood educators, and early interventionists in that providing early exposure to learning environments for behaviors relating to ATL may have a long-term positive impact on autistic children’s working memory as well as overall school readiness and adjustment. For instance, the Head Start and Early Head Start program, the largest early intervention and prevention program in the United States for low-resourced children from birth to 5 years, promotes school success by providing comprehensive services including educational, social, and health interventions for children with or without disabilities (Bratton et al., 2013). Such a program can be particularly beneficial for working memory development of autistic children from a low SES background if it is enriched with opportunities to acquire skills relating to ATL from a very early age.

Moreover, this study revealed that positive STR can be particularly beneficial for autistic children during their later years in elementary school. In contrast, a meta-analysis recently reported that the positive relationship between STR and working memory for the general population is stronger in early elementary school years than it is in later years (Vandenbroucke et al., 2018). It is unclear why there is a disparity between autistic children and the general population in the time period where STR predicts greater growth in working memory. However, a recent study indicated that autistic children who started kindergarten with poor working memory were more likely to make rapid growth during the latter half of their elementary school years, suggesting the presence of “late-bloomers” in working memory development in autistic children (Kim, 2022). Taken together, particularly for children on the autism spectrum, building strong STR can have a longitudinal benefit during the latter half of their elementary school years, especially for those who struggled with working memory during their early childhood. As prior studies indicate that children with autism have a more difficult time building a positive relationship with their teachers when compared to their neurotypical peers or peers with different disabilities (Blacher et al., 2014; Prino et al., 2016), careful attention must be paid to establishing high-quality STR with autistic children. Another meta-analysis found that positive STR can be fostered through universal school-wide programs that utilize proactive and direct measures (Kincade et al., 2020). Examples of such programs include Establish-Maintain-Restore (Cook et al., 2018), the BRIDGE program (Cappella et al., 2012), and the Incredible Years^®^ Teacher Management Program (Webster-Stratton et al., 2011). School districts’ collective efforts to implement such programs systematically and universally can support teachers to build positive relationships with their students, which in turn can benefit autistic students’ working memory development as well as other various school outcomes.

### Limitations and future directions

A few limitations must be noted in this study. While some covariates (e.g. SES, STR, ATL) can vary over time and can therefore exert a time-specific effect on autistic children’s working memory trajectories, composite scores were created and they were treated as time-invariant covariates. Although this decision was made for better interpretability and parsimony of the statistical models, time-specific effects were not captured in the study. More specifically, possible changes within these covariates across time points (e.g. changes in family income, different teachers at different time points) can impact the composite score, and therefore influence working memory development differently at varying time points. Therefore, it will be worthwhile to investigate the effects of one covariate at a time while treating the covariate as time variant. That way, we can acquire a deeper understanding of how each covariate influences children’s working memory trajectories over time. Furthermore, although the STRS (Pianta, 2001) contained both Closeness and Conflict scales, only Closeness scale was analyzed in the study. As research shows that children on the autism spectrum experience heightened conflicts with their teachers when compared to nonautistic peers (Blacher et al., 2014), future studies must also examine the roles of teacher-reported conflict in autistic children’s cognitive development. In addition, this study purposely examined a sample of autistic children only, in order to add to the existing literature base that explored contributing factors for EF development in neurotypical children. Therefore, the significant relationships found cannot be claimed as being specific to autistic children only. Future studies should examine if such relationships also exist in children with other developmental disabilities such as ADHD, or children with both autism and ADHD, in order to better understand the uniqueness of children’s EF development for each group. It is also important to note that selection of covariates in the analysis was limited to the set of measures included in the ECLS-K data set. Therefore, these contributing factors should not be interpreted as exclusive, as there may be other important factors that were not explored in this study. Furthermore, students in the sample may have participated in various educational (e.g. tutoring, after-school programs) or therapeutic services (e.g. in-home applied behavior analysis (ABA) services) outside of their typical school program, which were not captured in the current analysis. It is important to note that participation in these programs may have influenced these children’s developmental trajectories in working memory.

Moreover, some practical recommendations made in the study (e.g. Head Start program, Part C of IDEA) are limited to the United States, and parallel resources that are available internationally must be explored for children on the autism spectrum residing globally.

Regardless, this study is the first longitudinal study to our knowledge that examined predictors for autistic children’s working memory rate of growth over the entire elementary school years. While working memory capacity is said to be significantly influenced by common genetic factors (Ando et al., 2001), and impairments in working memory are common characteristic features of autism (Englund et al., 2014), findings from this study direct our attention to various environmental-level factors that could be arranged or manipulated. Results suggest that student demographic characteristics, learning behaviors, and school or home environment collectively play an important role in autistic children’s working memory development, and these are especially malleable during their elementary school years. Future studies should examine ways to maximize exposure to factors that were found to predict greater growth in working memory during developmentally sensitive periods of times for autistic children during elementary school.
